# Paraneoplastic acral vascular syndrome in a patient with metastatic melanoma under immune checkpoint blockade

**DOI:** 10.1186/s12885-017-3313-6

**Published:** 2017-05-12

**Authors:** Thilo Gambichler, Stefanie Strutzmann, Andrea Tannapfel, Laura Susok

**Affiliations:** 1 Department of Dermatology, Ruhr-University Bochum, Gudrunstr. 56, 44791 Bochum, Germany; 2 Institute of Pathology, Ruhr-University Bochum, Bürkle-de-la-Camp-Platz 1, 44789 Bochum, Germany

**Keywords:** Melanoma, Digital ischemia, Gangrene, Immune-checkpoint blockade, ipilimumab, nivolumab

## Abstract

**Background:**

Paraneoplastic acral vascular syndrome (PAVS) is a rare phenomenon which is observed in patients with adenocarcinomas and other malignancies. Various potential pathogenic mechanisms such as tumour invasion of sympathetic nerves, hyperviscosity, hypercoagulability, vasoactive tumour-secreted substances, and immunological mechanisms have been suggested.

**Case presentation:**

We report a 60-year-old Caucasian male attended our hospital with a bulky lymph node mass in the right axilla. Extirpation of a lymph node conglomerate revealed 5 melanoma lymph node metastases. Computed tomography showed a liver metastasis (diameter: 3.8 cm), several retroperitoneal metastases, bilateral metastases in the lung hilus, and prepectoral subcutaneous metastases (Stage IV; pTx, N3, M1c). Lactate dehydrogenase and S100B were slightly elevated. Combination therapy of nivolumab (1 mg/kg BW) and ipilimumab (3 mg/kg BW) was started. Three weeks after the first combination therapy he developed progressive erythema, paraesthesia and pain on the fingertips of both hands. Both cold and warmth was not well tolerated by the patient. Complete work-up excluded associated conditions or factors such as haematological disorders, rheumatologic disorders, hypertension, diabetes or smoking. Treatment was initiated with prostacyclin 20 μg twice daily and oral prednisolone 50 mg in tapering dosage. However, prostacyclin was stopped after the first applications because the pain increased during infusion. The second course of nivolumab and ipilimumab was administered. About 2 weeks later, the patient presented with increased pain and small subungual necrosis. We treated the patient with oral analgetics and intravenous prednisolone 500 mg in tapering dosage. On digital substraction angiography occlusion of all arteries of the fingers was demonstrated. Further rheologic and anti-melanoma treatments were refused by the patient. About 2 months after the second course of nivolumab and ipilimumab combination therapy several fingers showed severe gangrene which finally led to amputations of end phalanges of several fingers. Histopathology did not reveal evidence for vasculitis or other primary vascular pathologies. During the following 2 months the patient experienced dramatic progress of his metastatic disease and finally died at multi-organ failure.

**Conclusion:**

Presence of rapidly progressive digital ischemia in an elderly patient with cancer should always raise clinical suspicion of a paraneoplastic phenomenon when other possible causes have been excluded. In patients treated with immune checkpoint inhibitors such as CTLA-4 and PD-L1 blockers PVAS-like events have not been reported so far. However, it is debatable whether immune checkpoint blockade may play a pathogenetic role in the development of PAVS in patients with malignancies.

## Background

Paraneoplastic acral vascular syndrome (PAVS) is a rare phenomenon which is observed in patients with adenocarcinomas and other malignancies. Clinically, PAVS is similar to Raynaud’s phenomenon. However, PAVS is characterized by the association with malignancy and a rapid course of disease very frequently resulting in gangrene of fingers. The pathogenesis of PAVS is unclear but immunological mechanisms have been discussed [1–8]. The immune-checkpoint inhibitors (ipilimumab, nivolumab, pembrolizumab etc.) are increasingly used as anti-cancer agents as more efficacies have been proved in multiple cancer species, such as melanoma, non-small-cell lung carcinoma and renal cell carcinoma. Nevertheless, the therapy is associated with immune-related adverse events (irAE) occuring in more than 60% of treated patients. The pathophysiology of irAE is considered similar to that of autoimmune diseases, wherein activated lymphocytes target self-antigens [9, 10]. Here we report a patient with occult metastatic melanoma, who developed severe digital ischemia with gangrene after two applications of immune-checkpoint inhibitor combination therapy.

## Case presentation

**Fig. 1:**
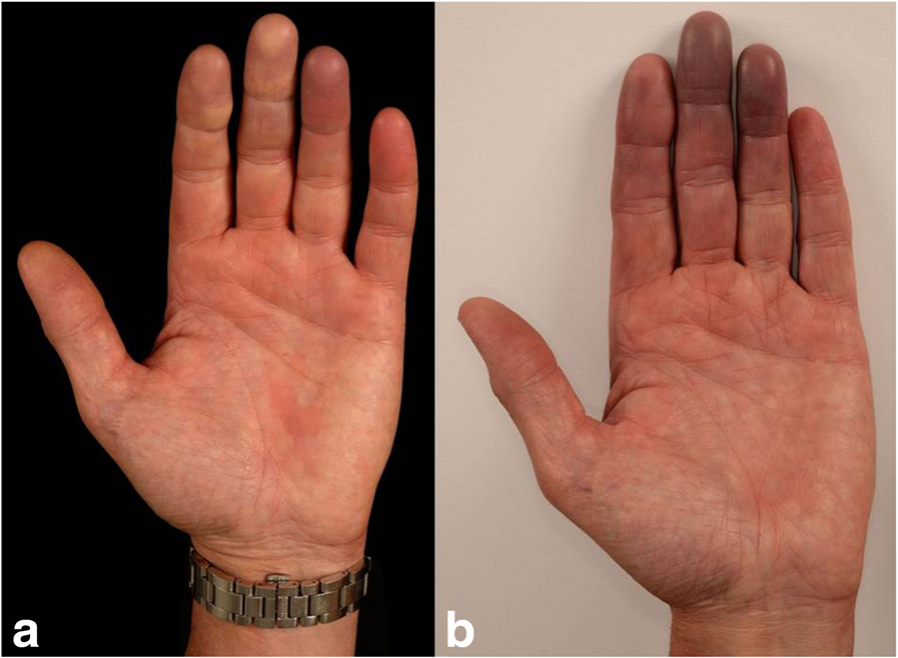
Twenty-five days after the first application of ipilimumab/nivolumab combination therapy the patient reported paraesthesia and pain in the fingertips; a slight erythema is visible on the fingertip of digitus 4 of the left hand (**a**). Five days after the second course ipilimumab/nivolumab combination therapy, a violaceous erythema was observed on digitus 3 and 4 of the left hand (**b**). Paraesthesia and pain also deteriorated

**Fig. 2:**
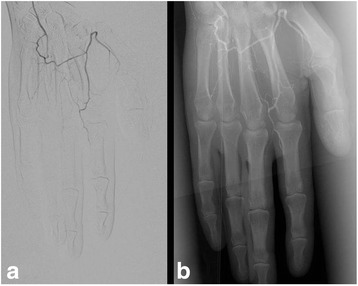
On digital substraction angiography an almost complete occlusion of digiti 2–5 was observed on the left hand (**a**). Nitroglycerin applications during angiography did not result in vasodilatation (**b**)

**Fig. 3:**
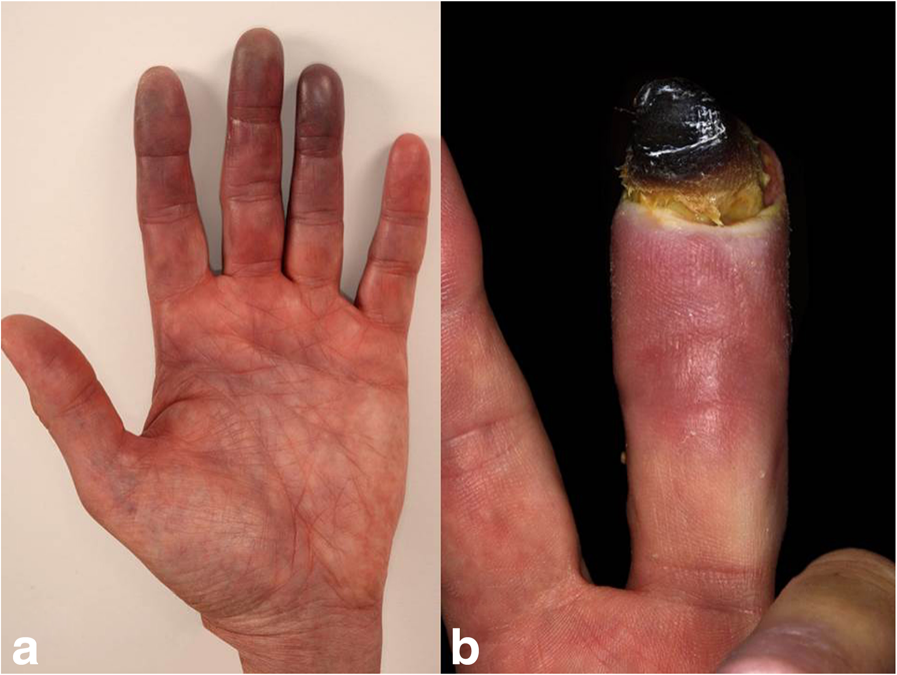
Twenty days after the second application of ipilimumab/nivolumab combination therapy the violaceous erythema was increased (**a**). The patient suffered from paraesthesia and strong pain. Six weeks later a severe gangrene was observed on digitus 3 of the left hand (**b**)

**Fig. 4:**
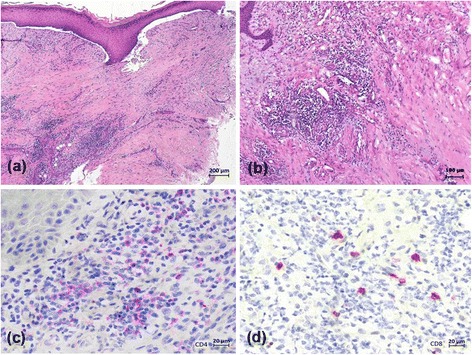
Histopathology of the skin assessed near the necrotic border of digitus 3 of the left hand (Fig. 3) predominantly revealed altered tissue with fibrosis and strong mainly perivascular inflammatory infiltrates including lymphocytes, plasma cells, and neutrophils (**a**, **b**). The inflammatory infiltrate was dominated by CD4+ cells (**c**) when compared to CD8+ lymphocytes (**d**)

We report a 60-year-old Caucasian male attended our hospital with a bulky lymph node mass in the right axilla. Extirpation of the lymph node conglomerate revealed 5 melanoma lymph node metastases - a primary melanoma was not found. Thoracic and abdominal computed tomography showed a liver metastasis (diameter: 3.8 cm), several retroperitoneal metastases, bilateral metastases in the lung hilus, and prepectoral subcutaneous metastases (Stage IV; pTx, N3, M1c; BRAF V600E mutated/KIT wildtype; ECOG = 0). Cranial magnetic resonance tomography did not reveal pathological findings. Lactate dehydrogenase (LDH) and S100B were slightly elevated with 357 U/I (135–225 U/I) and 0.38 μg/l (cut-off: 0.11 μg/l), respectively. According to the tumour board recommendation, combination therapy of nivolumab (1 mg/kg BW) and ipilimumab (3 mg/kg BW) was started after having performed electrocardiography and extensive lab investigations. Beside, slight elevation of the TSH receptor antibody, no relevant pathology was detected. Three weeks after the first combination therapy he developed slight erythema, paraesthesia and pain on the fingertips of both hands **(**Fig. 1 **)**. Ten days later, paraesthesia had worsened and livid erythema was observed in particular on digitus 2 and 3 of both hands. Both cold and warmth was not well tolerated by the patient. He had no prior history of trauma, cardiovascular illnesses, snake-bite, haematological disorders, rheumatologic disorders, hypertension, diabetes or smoking. He gave no history of having visited very high altitudes, or being exposed to very low temperatures which could have caused frostbite. On examination there were no clinical signs for rheumatic diseases such as systemic sclerosis (e.g., skin hardening, tightening of the facial skin, telangiectases, decreased oral aperture, and sicca-syndrome). Duplex-sonography of the hand arteries was unremarkable. Nail fold capillaroscopy did not demonstrate pathological findings. Lab tests including antithrombin III, fibrinogen, protein S and C did not reveal pathologies. Blood test was also negative for antinuclear antibodies, antineutrophil cytoplasmic antibody, rheumatoid factor, and cryoglobulins, and hepatitis (B and C) serology. Immunoglobulins and circulating immune complexes were within the normal range. Antiphospholipid and anticardiolipin antibodies (both IgG and IgM) and platelet count were within normal limits. Treatment was initiated with intravenous alprostadil 20 μg twice daily and oral prednisolone 50 mg in tapering dosage. However, alprostadil was stopped after the first applications because the pain increased during infusion. The second course of nivolumab and ipilimumab was administered. About 2 weeks later, the patient presented with progressive pain and small subungual necrosis. We treated the patient with oral analgetics and intravenous prednisolone 500 mg in tapering dosage. Combination immunotherapy was discontinued. On digital substraction angiography occlusion of all arteries of the fingers was demonstrated **(**Fig. 2 **)**. Several nitroglycerin applications during angiography did not favor a functional cause of ischemia. Further rheologic treatments (ilomedine, nifedipine etc.) and targeted therapy with a combination of a BRAF and MEK inhibitor was refused by the patient. About 2 months after the second course of nivolumab and ipilimumab combination therapy several fingers showed severe gangrene **(**Fig. 3 **)**. Amputations were finally performed on end phalanges of the right digitus III and left digiti III and IV. Histopathology predominantly revealed beside normal skin altered tissue with strong inflammatory infiltrates including lymphocytes, plasma cells, and neutrophils. The CD4/CD8 ratio was 3:1 (Fig. 4). Moreover, severe fibrosis and necrosis was observed. However, there was no evidence for vasculitis or other primary vascular pathologies. During the following 2 months the patient experienced dramatic progress of his metastatic disease (LDH > 3000 U/I) and finally died at multi-organ failure.

## Discussion and conclusion

PAVS including paraneoplastic Raynaud’s phenomenon or digital necrosis associated with malignancies comprise an uncommon clinical entity characterized by ischemia and necrosis predominantly affecting the hands. The underlying malignancies among patients with PAVS can be highly varied, such as carcinomas of the lung, stomach, breast, ovary, testes, and thyroid. Melanoma has very rarely been reported in the aforementioned context [1, 2, 8]. Skin lesions of PAVS usually appearing on the fingertips of both hands precede cancer in about half of cases. PAVS is more frequently seen in older male patients. The onset of PAVS occurs over a short time period and approximately 80% of patients rapidly develop gangrene [1, 2, 8]. Differential diagnosis particularly include Raynaud’s phenomenon which is a common condition occurring in up to 10% of the general population [11]. It is characterized by episodes or paroxysms of digital vasospasm in the hands and/or feet. These symptoms can frequently be triggered or aggravated by exposure to cold, emotional stress or vibrations. A typical episode of this vasogenic phenomenon encompasses a sequence of initial pallor, subsequent cyanosis followed by rubor and dolor (redness and pain). Up to 90% of all cases of Raynaud’s phenomenon can be classified idiopathic or primary - no underlying illness can be linked with [1, 2, 8, 11]. Among the aetiologies of secondary Raynaud’s phenomenon, the predominant aetiology would lie among the large spectrum of connective tissue and autoimmune diseases including scleroderma, mixed connective tissue disorder and rheumatoid arthritis [1, 2, 8, 11]. In contrast to Raynaud’s phenomenon [11], however, PAVS shows a sudden onset and a rapid progression of ischemia much more frequently resulting in gangrene of several fingers after a relatively short time. Moreover, PAVS is not necessarily associated with the classic episodes of vasogenic phenomenon as described above for Raynaud’s phenomenon. Another prominent cause of digital ischemia, especially in the younger male chronic smoker, would be thromboangiitis obliterans. Other causes of digital ischemia include frost-bite, vibrational trauma, snake-bite, hypothenar-hammer syndrome, peripheral atherosclerosis, hyperviscosity syndromes such as cryoglobulinaemia. The clinical presentation, patient’s history and complete work-up widely excluded the aforementioned differential diagnoses of PVAS in our patient [1, 2, 8, 11].

The pathomechanism of PAVS is unclear. Various potential pathogenic mechanisms such as tumour invasion of sympathetic nerves, hyperviscosity, hypercoagulability, vasoactive tumour-secreted substances, generalized vasospasm, and spontaneous platelet aggregation have been discussed [2]. Moreover, an immunological pathogenesis has been suggested. Numerous autoimmune phenomena are known to occur in malignancies including the detection of antinuclear antibodies and cryoglobulines [1, 8]. In the present case, an immunologic mechanism is likely since PAVS occurred after the initiation of an immune checkpoint blockade using ipilimumab and nivolumab. Notably, vascular events, including Raynaud’s phenomenon and even gangrene, have been observed in patients who underwent immunotherapy with alpha interferon [12]. Interestingly, interferon induced microvascular injury and endothelial cell damage has been reported in a mouse model [13]. In patients treated with immune checkpoint inhibitors such as cytoCTLA-4 and PD-1 blockers PVAS-like events have not been reported to our best knowledge [9, 10]. However, arteritis temporalis has sporadically been observed in melanoma patients treated with immune checkpoint inhibitors [9, 10]. Yshii et al. [14] recently demonstrated in a mouse model that the induction of a paraneoplastic neurological disorder after therapeutic blockade of CTLA4, raising the concern that checkpoint inhibitors, by enhancing anti-tumour immunity, may inadvertently increase the likelihood for paraneoplastic neurological disorders in which so-called onconeural antigens expressed by normal neurons and tumor cells play a crucial role [14]. We can only speculate whether, for example, endothelial antigens may have played a pathogenetic role in the present patient with PAVS and immune checkpoint blockade. On histopathology, however, there was no frank evidence for an immune complex- or cytotoxic T lymphocyte-related pathomechanism. With PAVS, benefit cannot be uniformly expected with modalities traditionally used in Raynaud’s phenomenon (nifedipine, surgical sympathetectomy, calcitonin, anticoagulants and prostacyclins). This could be because of the heterogeneity of possible underlying pathomechanisms in PAVS [1, 2, 8]. However, various reports have demonstrated that treatment of the malignancy also causes improvement with regards to the PAVS in about the half of cases [1, 2, 8].

In conclusion, the presence of PAVS in an elderly patient with cancer should always raise clinical suspicion of a paraneoplastic phenomenon when other possible causes have been excluded. It is debatable whether immune checkpoint blockade may play a pathogenetical role in the development of PAVS in patients with malignancies.

## Abbreviations

CTLA-4: Cytotoxic T lymphocyte antigen-4
irAE: Immune-related adverse events
LDH: Lactate dehydrogenase
PAVS: Paraneoplastic acral vascular syndrome
PD-1: Programmed death receptor-1
